# Study liquid–liquid phase separation with optical microscopy: A methodology review

**DOI:** 10.1063/5.0137008

**Published:** 2023-05-09

**Authors:** Xiufeng Zhang, Haoyang Li, Yue Ma, Dongping Zhong, Shangguo Hou

**Affiliations:** 1 School of Chemistry and Chemical Engineering, Shanghai Jiao Tong University, Shanghai 200240, China; 2 Institute of Systems and Physical Biology, Shenzhen Bay Laboratory, Shenzhen 518055, China; 3 State Key Laboratory of Precision Spectroscopy, East China Normal University, Shanghai 200062, China; 4 Center for Ultrafast Science and Technology, School of Chemistry and Chemical Engineering, Shanghai Jiao Tong University, Shanghai 200240, China; 5 Department of Physics, Ohio State University, Columbus, Ohio 43210, USA; 6 Department of Chemistry and Biochemistry, Ohio State University, Columbus, Ohio 43210, USA

## Abstract

Intracellular liquid–liquid phase separation (LLPS) is a critical process involving the dynamic association of biomolecules and the formation of non-membrane compartments, playing a vital role in regulating biomolecular interactions and organelle functions. A comprehensive understanding of cellular LLPS mechanisms at the molecular level is crucial, as many diseases are linked to LLPS, and insights gained can inform drug/gene delivery processes and aid in the diagnosis and treatment of associated diseases. Over the past few decades, numerous techniques have been employed to investigate the LLPS process. In this review, we concentrate on optical imaging methods applied to LLPS studies. We begin by introducing LLPS and its molecular mechanism, followed by a review of the optical imaging methods and fluorescent probes employed in LLPS research. Furthermore, we discuss potential future imaging tools applicable to the LLPS studies. This review aims to provide a reference for selecting appropriate optical imaging methods for LLPS investigations.

## I. INTRODUCTION

Cellular compartmentalization ensures that various essential cellular biochemical activities are carried out in an orderly, efficient, and precise manner. Classical membrane-enclosed compartmentalizations, such as mitochondria, Golgi apparatus, endoplasmic reticulum, and cell nucleus chloroplasts, play crucial roles in this process.^1^ Liquid–liquid phase separation (LLPS) represents another mechanism that facilitates the formation of membraneless organelles (MLOs) necessary for a wide range of functions. The concept of LLPS can be traced back to 1899 when Edmund B. Wilson proposed that the cytoplasm behaves like liquid emulsions.^2^ In 2009, the first condensates (P particles) were discovered *in vivo*.^3^ Subsequently, LLPS condensates have been observed in the nucleus,^4–6^ cytoplasm,^7–9^ chloroplasts,^10,11^ and at the membrane surface,^12–14^ highlighting their importance in cellular organization and function.

**FIG. 1.**
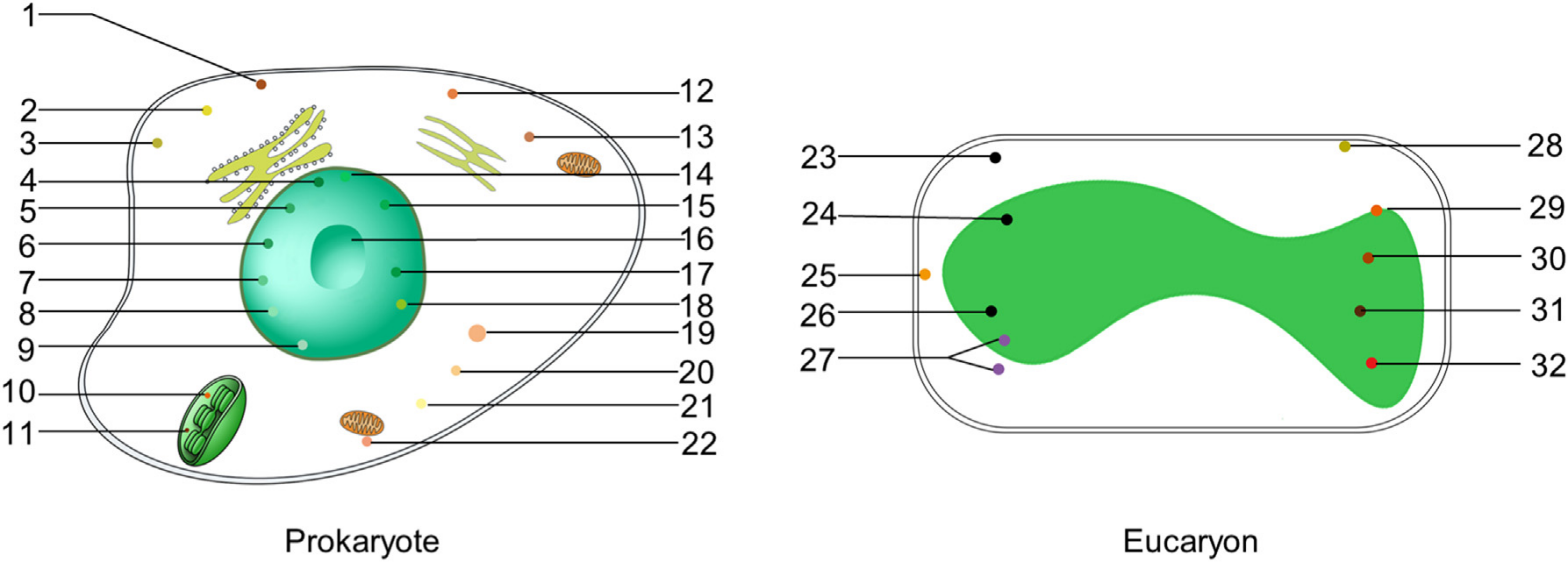
LLPS condensates in eucaryon and prokaryotes. 1, NONEXPRESSOR OF PATHOGENESIS-RELATED GENES 1(NPR1) body; 2, Stress granule; 3; Processing body; 4, Cajal body; 5, DNA damage body; 6, PcG body; 7, ELF3 body;8, Dicing body; 9, FLOWERING LOCUS CA (FCA) body; 10, Pyrenoid matrix; 11, STT1/STT2; 12, ATE EMBRYOGENESIS ABUNDANT(LEA)body; 13, AUXIN RESPONSE FACTOR (ARF) body; 14, Photobodies; 15, Nuclear speckles; 16, Nucleolus; 17, Histone locus body; 18, Paraspeckle; 19, Germ granule; 20, Balbiani body; 21, RNA transport granule; 22, Mitochondria-associated ribonucleoprotein domain (MARDO); 23, Ribonucleoprotein bodies(BR-bodies); 24, RNA polymerase clusters(RNAP clusters); 25, PopZ microdomains; 26, Nucleoid protein Dps; 27, Single-stranded DNA-binding protein(SSB); 28, ATP-binding cassette (ABC)transporter Rv1747; 29, Divisome protein FtsZ and SlmA; 30, Polyphosphate (polyP)granules; 31, ParB-parS cluster; 32, Carboxysome.

LLPS is intimately linked to the pathogenesis of various diseases, including neurodegenerative disease such as amyotrophic lateral sclerosis (ALS),^15,16^ frontotemporal dementia (FTD),^17,18^ Alzheimer's disease (AD),^19,20^ and Parkinson's disease (PD).^21^ It is also associated with solid tumors, including breast cancer,^22^ colon cancer (CC),^23^ hepatocellular carcinoma (HCC),^24^ SARS-CoV-2 infection,^25–27^ and leukemia.^28^ In plants, LLPS plays a role in light response,^29–31^ heat response,^31,32^ hormone signaling,^33^ and CO_2_ response.^34^ In bacteria, LLPS is related to fitness^35^ and antibiotic tolerance.^36^ Therefore, LLPS appears to be a ubiquitous phenomenon in both prokaryotic and eukaryotic cells (Fig. 1).

Common research techniques in LLPS studies include computational approach using sequence analysis (to identify LLPS protein),^37^ fluorescence recovery after photobleaching(FRAP),^38–42^ fluorescence correlation spectroscopy (FCS)^43^ (to calculate material exchange between LLPS condensates and their surroundings), live cell optical morphology imaging (to observe droplet fusion or separation),^23,32^ and *in vitro* remodeling (to explore phase separation structures that can be reconstructed using purified proteins under physiologically relevant conditions).^32,44^ Several excellent reviews have discussed LLPS mechanisms and their roles in cellular biology and physiology.^44–50^ In this review, we focus on the optical imaging methods applied to LLPS studies. We begin with a brief introduction to the molecular mechanism of LLPS, followed by a review of the optical imaging methods and fluorescent probes used in LLPS studies. Finally, we discuss emerging imaging techniques that could be applied to LLPS studies in the future.

## II. MOLECULAR MECHANISM OF LIQUID–LIQUID PHASE SEPARATION

**FIG. 2.**
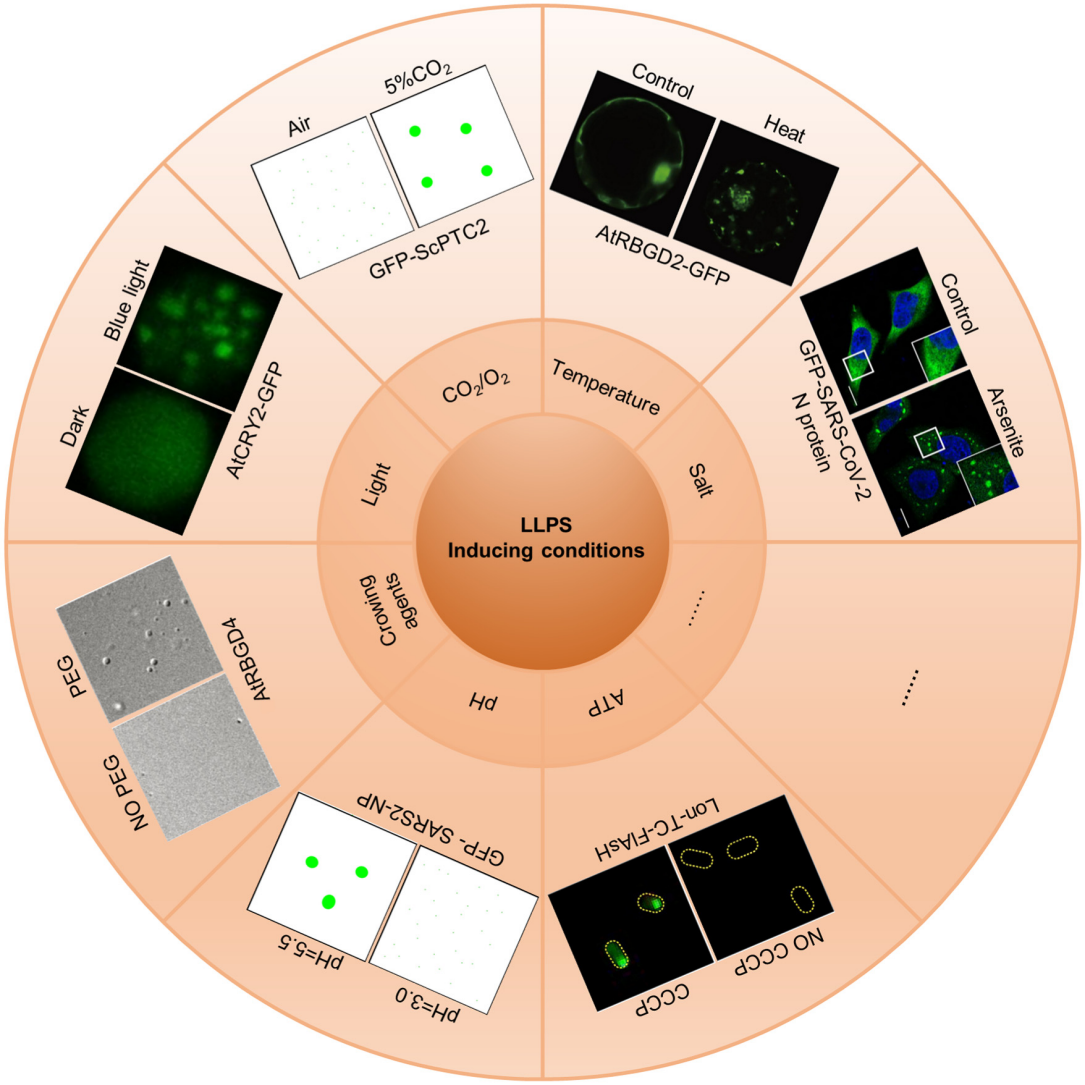
LLPS inducing conditions. Salt: GFP-tagged SARS-CoV-2 nucleocapsid proteins form droplets upon arsenite stimulation in HeLa cells [Reproduced with permission from Wang *et al.*, Cell Discovery **7**(1), 5 (2021). Copyright 2021 Authors, licensed under a Creative Commons Attribution 4.0 International License^54^]; temperature: AtRBGB2–GFP proteins form dynamic granule-like structures after heat treatment in *Arabidopsis* protoplasts [reproduced with permission from Zhu *et al.*, Dev. Cell **57**(5), 583–597 (2022). Copyright 2022 Elsevier^32^]; CO_2_/O_2_: the cartoon of condensate formation of GFP-ScPtc2 is induced under 5% CO_2_; Light: AtCRY2–GFP proteins form photobodies in *Arabidopsis* protoplasts under blue light [Reproduced with permission from Mo *et al.*, Nat. Commun. **13**(1), 2631 (2022). Copyright 2022 Authors, licensed under a Creative Commons Attribution 4.0 International License^30^]; crowding agents: PEG is used to induce LLPS of AtRBGD4 *in vitro* [Reproduced with permission from Zhu *et al.*, Dev. Cell **57**(5), 583–597 (2022). Copyright 2022 Elsevier^32^]; pH: the cartoon of GFP-SARS2-NP proteins form droplets under pH jumping from 3.0 to 5.5 *in vitro*; ATP: Lon-TC-FlAsH protein aggregates are induced by CCCP used for ATP depletion [Reproduced with permission from Pu *et al.*, Mol. Cell **73**(1), 143–156 (2019). Copyright 2018 Elsevier^36^].

**FIG. 3.**
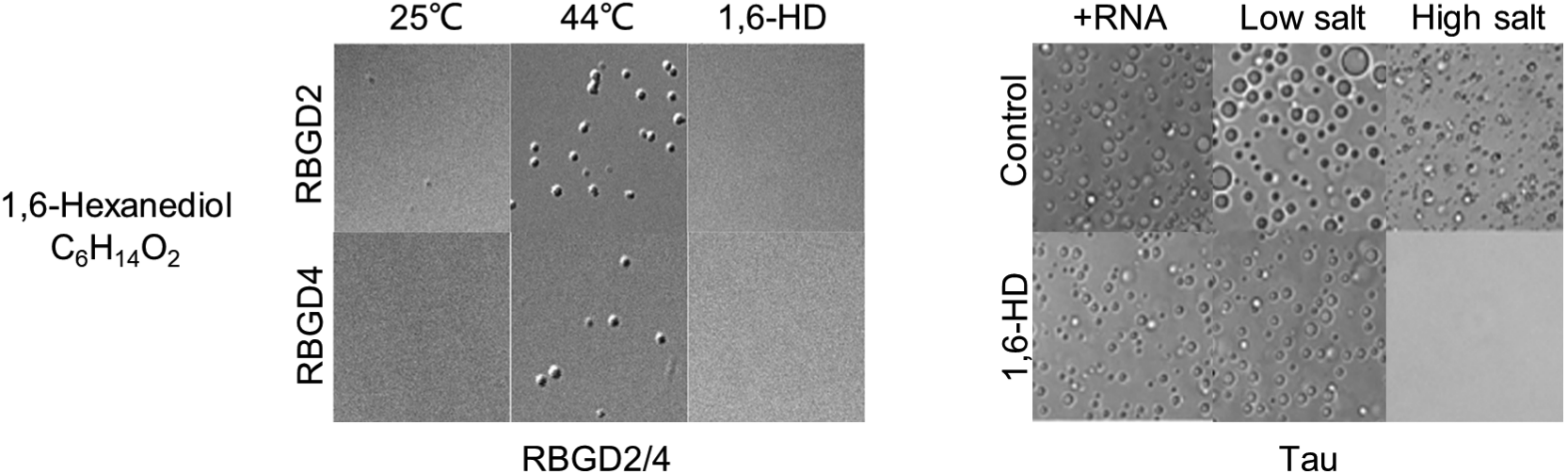
RGBD2/4 [Reproduced with permission from Zhu *et al.*, Dev. Cell **57**(5), 583–597 (2022). Copyright 2022 Elsevier^32^] and Tau [Reproduced with permission from Lin *et al.*, J. Mol. Biol. **433**(2), 166731 (2021). Copyright 2021 Elsevier^55^] condensates are both disrupted by 1,6-HD treatment.

Proteins involved in LLPS undergo a transition between a monodisperse state and a droplet state, influenced by various environmental factors such as salt concentration, temperature, CO_2_/O_2_ levels, light exposure, crowding agents, pH, and ATP, among others (Fig. 2). The formation of larger droplets from smaller ones can be facilitated by fusion events and/or Ostwald ripening.^51^ In the droplet state, there is a rapid exchange of molecules between the droplet and its surroundings.^39,41^ However, as droplets age, they transition to form gel-like structures or Maxwell glasses, where the exchange rate slows down.^20^ The primary driving forces behind LLPS include electrostatic and hydrophobic interactions such as cation–anion, cation–π, π–π, dipole–dipole, helix–helix, coiled-coil, steric zipper, and oligomerization.^52^ For fused in Sarcoma (FUS) in LLPS, the π-cation interaction serves as the major driving force and can be modulated by ATP and oligonucleotides.^53^ ATP functions not only as an energy storage molecule but also as an inducer of LLPS.^35^ Aliphatic alcohol 1,6-hexanediol (1,6-HD) is commonly used to inhibit the formation of LLPS condensates both *in vitro* and within cells by disrupting the hydrophobic interactions among proteins (Fig. 3).

According to PhaSepDB2.1, a database dedicated to phase-separation-related proteins (http://db.phasep.pro/), 868 proteins are associated with LLPS.^56^ Proteins that undergo LLPS typically feature either well-folded, repetitive modular domains composed of a series of similar or identical structural units or intrinsically disordered regions (IDRs). These domains play a crucial role in promoting LLPS formation by enabling multivalent intermolecular interactions. Low complexity domains (LCDs) are a distinctive feature of IDRs, as they lack a rigid tertiary structure due to the absence of hydrophobic amino acids.^57,58^ Moreover, post-translational modifications (PTMs) in IDRs, such as Serine/Threonine/Tyrosine phosphorylation, Arginine Methylation, Arginine Citrullination, Lysine Acetylation, and Poly(ADP-Ribosylation), significantly contribute to LLPS formation.^59^

LLPS plays a role in various biological processes, including relegating the concentration of intracellular proteins, activating signal transduction pathways, activating biochemical reactions, sensing environmental stimuli, and mediating protein localization.^45,60^ Tau, a microtubule-associated protein, is a key histopathological signature protein involved in many neurodegenerative diseases.^61^ Recent research indicates that Tau protein can form condensates by itself at high concentrations, with LLPS formation modulated by factors like Zinc,^62^ myricetin,^63^ RNA,^64^ and RNA binding protein TIA1.^65^ Under normal physiological conditions, tau is mainly involved in regulating synaptic plasticity, modulating neuronal activity, and participating in signal transduction pathways.^66^ However, under pathological conditions, tau protein aggregation occurs, leading to neuronal dysfunction.^67,68^

LLPS is essential for mRNA storage during transcription pausing in nuclear condensation of spermatids and the final stages of oocyte development, ensuring successful reproduction and development. During male germ development, the RNA-binding protein FXR1 can recruit spermiogenic mRNA to form messenger ribonucleoproteins (mRNPs) by LLPS, while in late spermatids, FXR1 activates the translation of mRNAs by recruiting translational machinery.^69^ Similarly, a mitochondria-associated membraneless compartment ribonucleoprotein domain (MARDO) can store translationally repressed mRNA by LLPS, which is important for oocyte maturation or early embryonic development.^70^

Furthermore, LLPS of the coronavirus SARS-CoV-2 nucleocapsid (N) protein and genomic RNA not only facilitates their assembly and transcription but also diminishes stress granule formation and suppresses the innate antiviral immune response in host cells.^27^ In leukemia, nucleoporin NUP98-HOXA9 condensates play a critical role in cancerous transformation and promote transcriptional activation of downstream oncogenic genes.^71^ Similarly, in colon cancer, Micro RNA (miR)-490-3p can silence its target Cyclin-dependent kinase 1 (CDK1) by LLPS, leading to G1/S arrest and inhibiting cell proliferation.^23^

## III. OPTICAL IMAGING METHODS IN LLPS STUDY

### A. Differential interference contrast (DIC) microscope

**FIG. 4.**
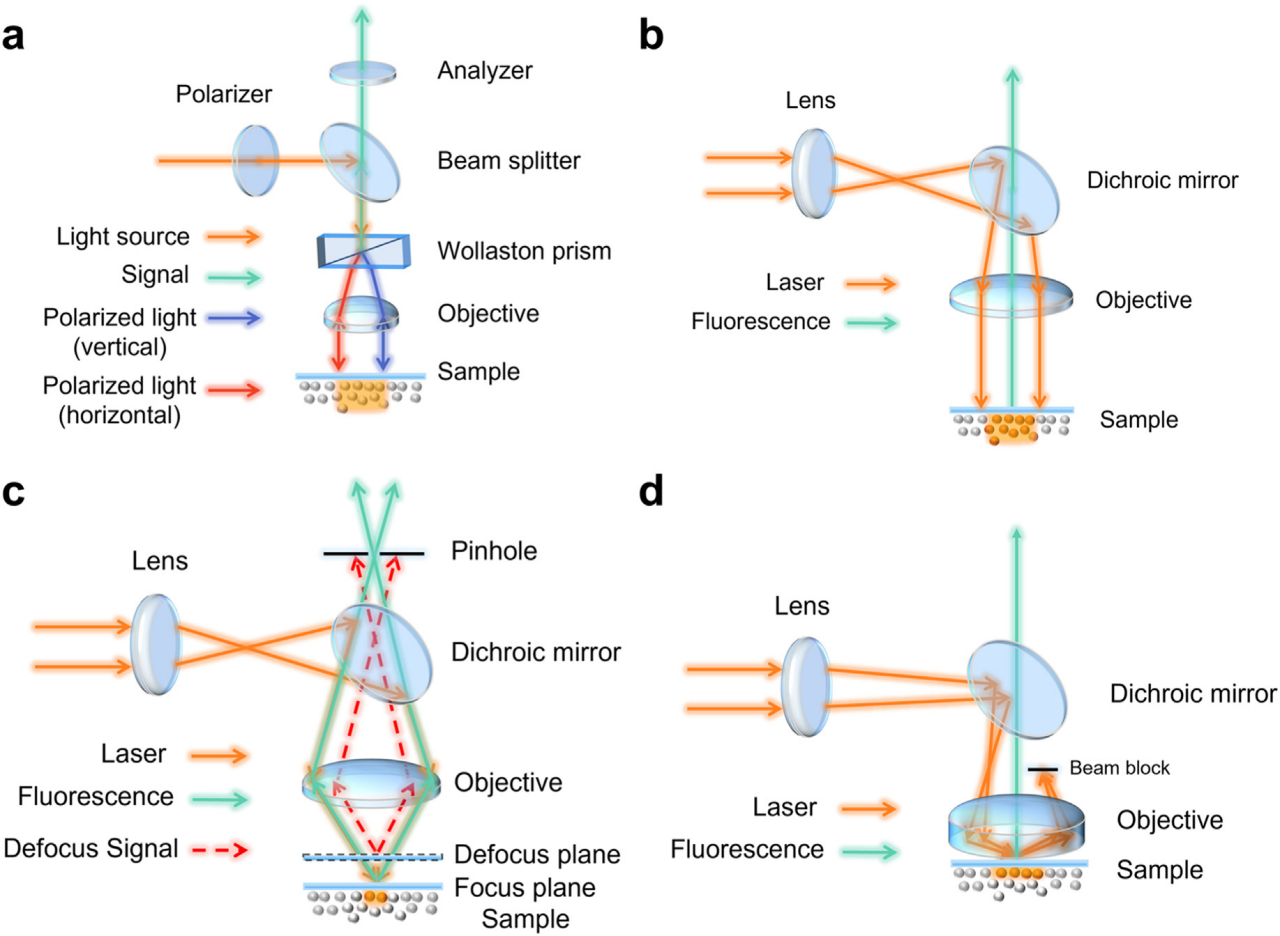
Schematics of the differential interference contrast microscope (a), wide-field fluorescence microscope (b), confocal microscope (c), and total internal reflection fluorescence microscope (d).

Differential interference contrast (DIC) is an imaging technique that converts the optical path gradient of a specimen into an amplitude difference image. A DIC microscope requires a polarizer, an analyzer, and a Wollaston prism, as illustrated in Fig. 4(a). In essence, linearly polarized light is divided by a Wollaston prism into two beams with mutually perpendicular polarizations, which then illuminate the sample. As the thickness and refractive index of the sample vary, the light paths of the two beams passing through the sample also change. The two signal beams reflected by the sample pass through the Wollaston prism and the analyzer. Due two beams scattering and reflection by the specimen, these two beams may experience different optical paths, resulting in interference and contrast in the specimen. DIC microscopy enables direct imaging of cells or samples with a refractive index gradient, without the need for fluorescent labels. In particular, droplets formed by liquid–liquid phase separation result from a the highly concentrated liquid phase and exhibit a well-defined, dynamic boundary with a distinct refractive index gradient from the solvent. A DIC microscope allows for rapid and convenient imaging of phase-separated droplets formed *in vitro*, making it widely used in the study of the properties, functions, and kinetics of LLPS *in vitro*.^20^,^72–79^

**FIG. 5.**
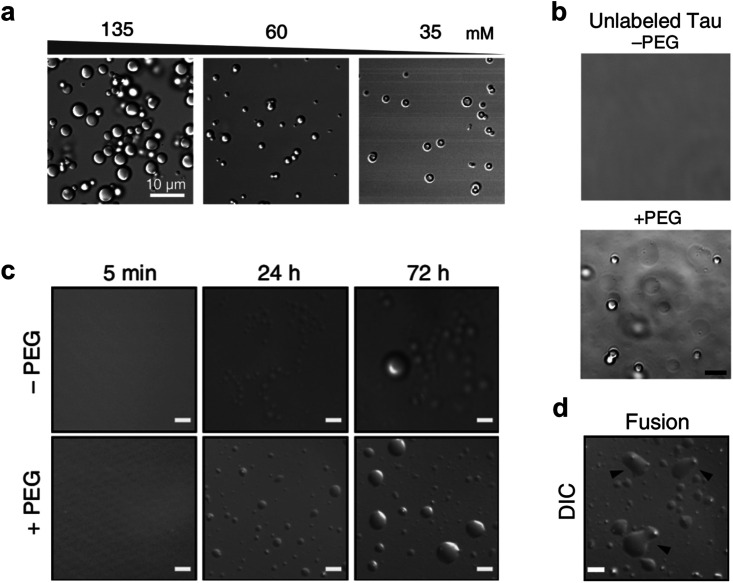
Study LLPS with the DIC microscope. (a) DIC images of the droplets formed by 20×CAG RNA in the presence of decreasing concentrations of Mg^2+^. Scale bar = 10 *μ*m. [Reproduced with permission from Ma *et al.*, J. Am. Chem. Soc. **144**(11), 4716–4720 (2022). Copyright 2022 J. Am. Chem. Soc.^75^] (b) DIC images of unlabeled full-length human tau forming phase-separated droplets, both in the absence (top) and presence (bottom) of molecular crowding agent PEG. Scale bar = 5 *μ*m. [Reproduced with permission from Kanaan *et al.*, Nat. Commun. **11**(1), 2809 (2020). Copyright 2020 Authors, licensed under a Creative Commons Attribution 4.0 International License.^79^] (c) DIC images of K18 forming phase-separated droplets in solution, both in the absence (top row) and presence (bottom) of molecular crowding agent PEG. Scale bar = 10 *μ*m. [Reproduced with permission from Ambadipudi *et al.*, Nat. Commun. **8**(1), 275 (2017). Copyright 2017 Authors, licensed under a Creative Commons Attribution 4.0 International License.^77^] (d) The K18 droplets (with PEG) in Fig. 5(c) undergo fusion. Scale bar = 10 *μ*m. [Reproduced with permission from Ambadipudi *et al.*, Nat. Commun. **8**(1), 275 (2017). Copyright 2017 Authors, licensed under a Creative Commons Attribution 4.0 International License.^77^]

Tang *et al.* studied the phase separation of RNA molecules *in vitro* using DIC microscopy and hyperspectral stimulated Raman microscopy.^75^ Their results revealed that RNA molecules containing 20× CAG repeats self-assemble into three droplets with different morphologies in the presence of Mg^2+^. The Mg^2+^ regulation of RNA droplets may be closely related to the general aging process of RNA-containing membrane-less organelles. As shown in Fig. 5(a), the morphology of RNA droplets changed gradually with the decrease in Mg^2+^ concentration. In addition, DIC microscopy is a common tool for exploring the mechanism of protein Tau phase separation *in vitro*.^20^,^77–79^ The study successfully captured the formation dynamics of K18 solution phase separation droplets under the influence of polyethylene glycol (PEG) [Fig. 5(c)]. PEG was observed to accelerate the fusion of K18 phase-separated droplets leading to the formation of larger droplets [Fig. 5(d)]. The full-length human Tau protein exhibited similar properties *in vitro*.^79^ As shown in Fig. 5(b), PEG promotes the formation of full-length human Tau protein phase separation droplets.

DIC microscopy is a valuable technique for studying the morphological changes of phase-separated droplets *in vitro*. It is particularly effective for characterizing LLPS due to its ability to provide higher contrast than bright field microscopy. This high contrast originates from the differences in refractive indices within the specimen. Phase-separated droplets are typically transparent, rendering small droplets undetectable under bright field microscopy. However, with DIC microscopy, these droplets exhibit better contrast because of the refractive indices difference. DIC microscopy lacks the specificity required to capture small LLPS droplets inside cells. The complexity of intracellular components necessitates the use of specific fluorescent labels to study intracellular biological events. Therefore, fluorescence microscopy is a more efficient tool for investigating the phase separation process in cells compared to DIC microscopy.

### B. Wide-field fluorescence microscope and confocal microscope

Fluorescence microscopy is a widely used technique that relies on autofluorescence or fluorescent tags to image samples. It enables the examination of cells or tissues that have been specifically labeled with fluorescent markers. The structure of fluorescence wide-field microscopy is demonstrated in Fig. 4(b). Briefly, the laser beam is focused by a lens onto the back focal plane of the objective. The beam is then reflected by the dichroic mirror into the objective lens, forming a parallel light field that irradiates the sample. The fluorescent signals of the samples are collected by the objective lens and detected after passing through the dichroic mirror. In previous works,^72,76^ fluorescence wide-field microscopy was used to study the formation or properties of LLPS droplets both *in vivo* and *in vitro*. Fluorescence microscopy provides several advantages for studying LLPS, including high controllability, adjustable environmental conditions, and good repeatability *in vitro*. Additionally, it offers intuitive visualization, non-invasiveness, high spatiotemporal resolution, and reversibility *in vitro*. However, due to the large illumination depth of wide-field fluorescence microscope, sample signals in out-of-focus areas are also collected, significantly reducing the signal-to-noise ratio of imaging. To solve this problem, confocal fluorescence microscopy imaging technology has been proposed.

**FIG. 6.**
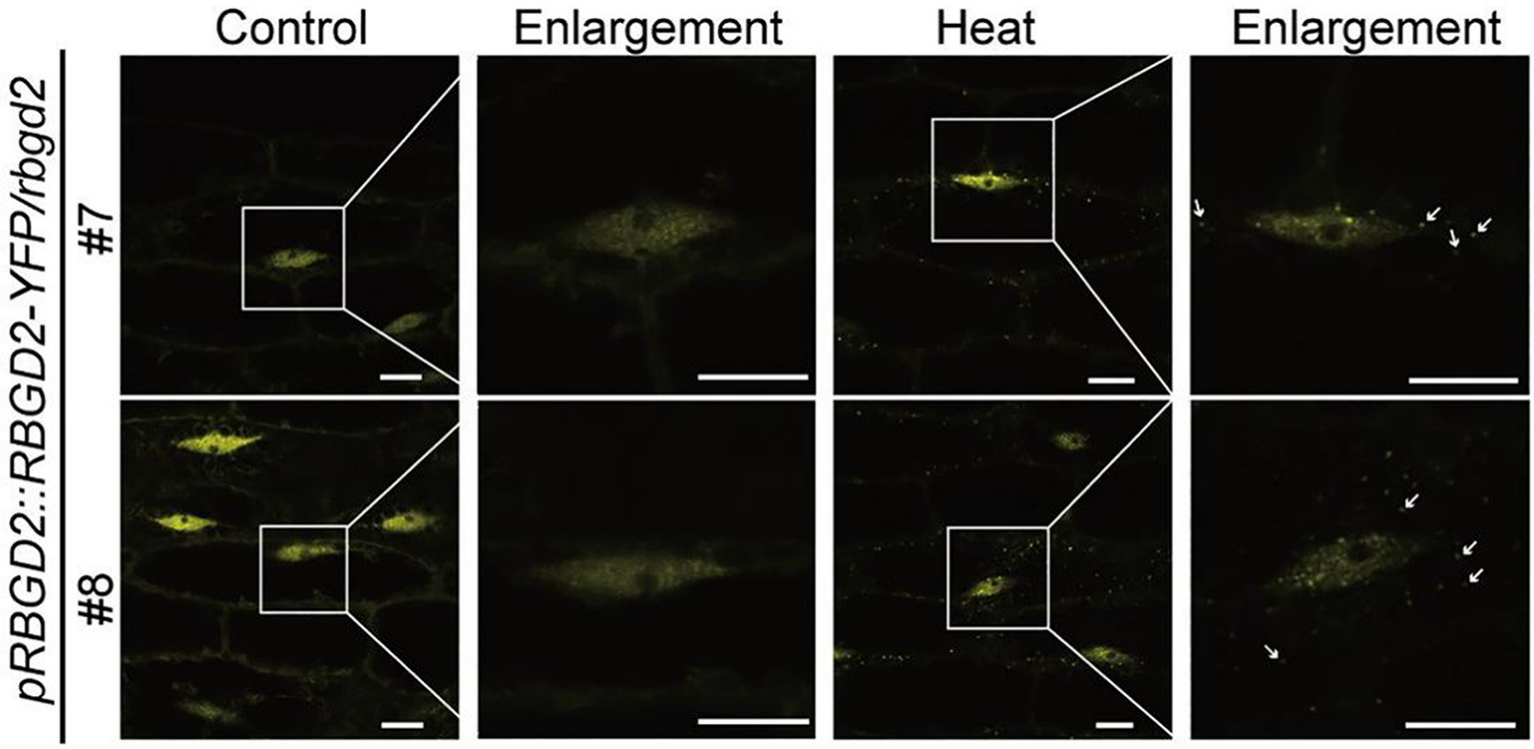
Study LLPS with the confocal microscope. After heat treatment, native promoter-driven RBGD2-YFP proteins driven form droplet-like structures. Scale bar = 10 *μ*m. [Reproduced with permission from Zhu *et al.*, Dev. Cell **57**(5), 583–597 (2022). Copyright 2022 Elsevier.^32^]

Over the past 30 years, confocal microscopy has become an important tool in biological studies. In confocal microscopy, the focused beam replaces the wide field beam as the excitation light source [Fig. 4(c)]. The signal is collected by the objective lens and passes through a pinhole before being detected. The excitation focus is conjugated with the detection pinhole, which serves as a spatial light filter to reject out-of-focus light and enhance the axial resolving power of confocal microscopy. Out-of-focus light is effectively attenuated, significantly improving the signal-to-noise ratio of imaging. The entire sample can be scanned by controlling the spot and recording the signal from each point, thus imaging a plane (focal plane). The rapid development of confocal microscopy technology, along with accompanying labeling techniques and image-processing methods over the past decades, has provided extremely important assistance for LLPS research. The formation of phase-separated droplets by tau protein *in vitro* or *in vivo* can be captured with high efficiency by confocal microscopy.^20,77,79^ Recently, Cheng *et al.* showed that mammalian oocytes can store mRNAs in mitochondria-associated membrane-less compartments.^70^ These membrane-less compartments, which are droplets formed by spontaneous phase separation of proteins and/or nucleic acids, play important roles in mRNA storage, translation, and degradation, and mammalian fertility ensuring. Kang *et al.* investigated the formation of phase-separated droplets by FXR1 in spermatids *in vivo* and *in vitro* using confocal microscopy.^69^ Their study indicates that these LLPS droplets critically contribute to the translational activation of stored mRNAs in mouse spermatids and male fertility in mice. Moreover, in Arabidopsis thaliana, CRY2 forms photobodies under blue light stimulation,^29^ and RBGD2/4 forms stress granules under temperature stimulation (Fig. 6). These bodies were all observed to have droplet-like structures and were confirmed to undergo phase separation using confocal microscopy.

Confocal microscopy can produce high-resolution images by finely sectioning biological samples. In particular, it is a powerful tool to study the formation or properties of LLPS. However, its limitations in acquisition speed render it unsuitable for imaging highly dynamic processes. The point excitation characteristic of confocal microscopy results in greater phototoxicity compared to wide-field illumination. Furthermore, achieving single-molecule level imaging with confocal microscopy is challenging. Therefore, capturing the dynamics at the early stage of phase separation droplet formation, such as the transition process from monomeric molecules to micrometer-scale phase separation droplets, remains a challenge for confocal microscopy.

To enhance acquisition speed and reduce phototoxicity, a modified version of the conventional confocal microscope known as the spinning disk confocal microscope has been developed.^80,81^ This microscope features a rotating turntable with pinholes arranged in an Archimedes spiral, placed at the object mirror plane, and a laser light source that covers the entire range of pinholes. As the turntable rotates, each pinhole scans a corresponding section of the sample, allowing for comprehensive scanning. This multipoint synchronous scanning technique significantly increases acquisition speed while reducing excitation light power, thus minimizing photobleaching and photodamage to the sample. With the spinning disk confocal microscope, studies of LLPS benefit from faster image acquisition rates, allowing for visualization of droplet formation, growth, and movement over time, as well as their interactions with other cellular components, at a higher temporal resolution than conventional confocal microscopy.^82^ However, this method sacrifices some spatial resolution in the images. Thus, researchers studying LLPS must rationally choose between the two imaging tools based on their research requirements. The spinning disk confocal microscope is ideal for higher temporal resolution data, while conventional confocal microscopy should be selected for higher spatial resolution data.

### C. Total internal reflection fluorescence (TIRF) microscopy

**FIG. 7.**
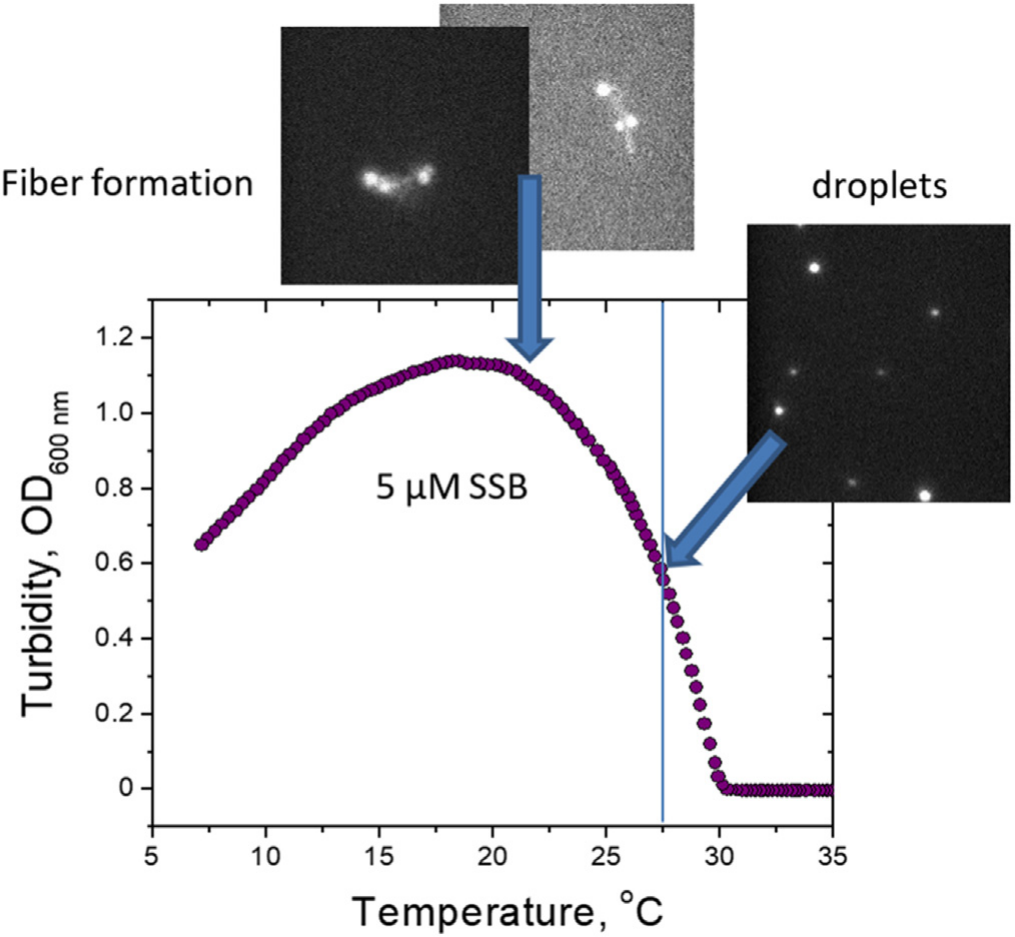
Study LLPS with TIRF-M. Microscopic observations support the formation of liquid phase droplets in the range of linear increase in turbidity and formation of solid phase at the maximum of turbidity curve. [Reproduced with permission from Kozlov *et al.*, J. Mol. Biol. **434**(9), 167562 (2022). Copyright 2022 Elsevier.^87^]

Total internal reflection fluorescence (TIRF) microscopy (TIRF-M) is a widely used optical technique in cell biology, particularly for analyzing cell membranes, studying the localization and dynamics of single molecules.^83–86^ In TIRF-M the excitation light is totally internally reflected at the interface between a transparent solid and a liquid, generating an evanescent wave—an electromagnetic field with the same frequency as the excitation light in the liquid at the solid–liquid interface.^83,86^ In brief, TIRF-M can induce an evanescent wave or field in a finite sample area immediately adjacent to the interface between two media with different refractive indices, with the excitation depth being less than 100 nm from the thickness of the solid surface [Fig. 4(d)]. This thin section property results in low background noise for TIRF imaging, and the adoption of electron-multiplying charge-coupled device (EMCCD) enables single molecule sensitivity in the TIRF microscope. TIRF-M is also easy to implement by changing the light path under a wide-field microscope, making it a popular choice for LLPS research. Maruri-López *et al.* utilized TIRF-M to study the LLPS of *Escherichia coli* single-stranded DNA-binding protein (EcSSB).^87^ They found that increasing [KGlu] in the physiological range promotes LLPS droplets, whereas increasing [KCl] and/or deleting IDL disables LLPS (Fig. 7). This indicates the importance of salt for the regulation of LLPS of EcSSB. Zhang *et al.* used TIRF microscopy to study the formation process of P62/SQSTM1,^88^ which is a selective autophagy receptor that mediates the formation of ubiquitinated proteins.

However, TIRF-M is limited to imaging near the surface, and the droplet size formed by LLPS can be smaller than the optical diffraction limit in the early stages. This makes it challenging to accurately characterize and quantify droplets using traditional fluorescence microscopes. Thus, the fluorescence microscope with higher resolution (super-resolution fluorescence microscope) should be used to study the characteristics and formation kinetics of LLPS.

### D. Super-resolution microscopy techniques

Biological events occurring at nanometer scales (5–100 nm) are of great interest for understanding biological dynamics, functions, and structures.^89^ However, the diffraction limit of optical microscopy limits the spatial resolution of fluorescence microscopy, making it difficult to visualize these events. To overcome this limitation, a series of super-resolution fluorescence microscopy techniques have been proposed since 1994.^90^ These techniques rely on sequentially positioning individual fluorophores by spatially or temporally modulating their fluorescence.^91,92^

**FIG. 8.**
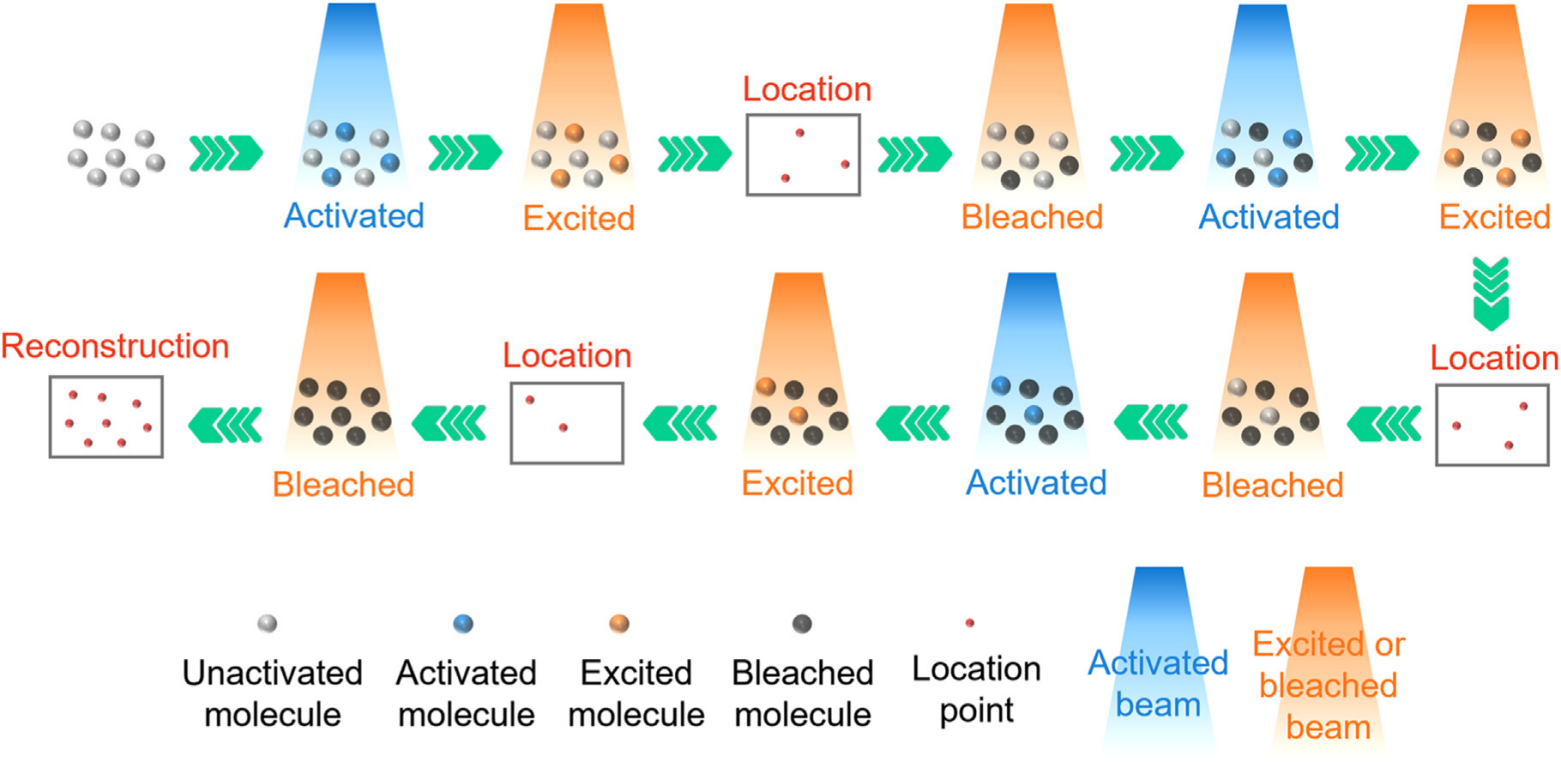
Schematic of PALM.

Photo-activated localization microscopy (PALM) and stochastic optical reconstruction microscopy (STORM) are two commonly used single molecule localization super-resolution microscopy (SMLM) techniques that have been applied in many biological studies.^93–96^ In PALM, a subset of inactive fluorescent groups is randomly activated by a weak short wave laser beam. To ensure that only a few fluorescent molecules are activated in the diffraction-limited area, it is necessary to control the activation beam to maintain sufficiently low intensity. These activated fluorescent molecules are then excited by another beam with a longer wavelength and higher intensity, and resulting the emission is collected. As the distance between excited fluorescent molecules is greater than the diffraction limit, it is easy to determine the center of the Airy disk and accurately locate the position of the fluorescent molecules. After the location information is obtained, these fluorescent molecules are photobleached. Another group of inactive fluorescent molecules is activated, excited, located, and photobleached. By repeating the above steps and recombining the information of all localized fluorescent molecules, a complete super-resolution image is generated (Fig. 8). PALM can be realized by a wide-field microscope or TIRF-M, and its engineering implementation cost is low.

**FIG. 9.**
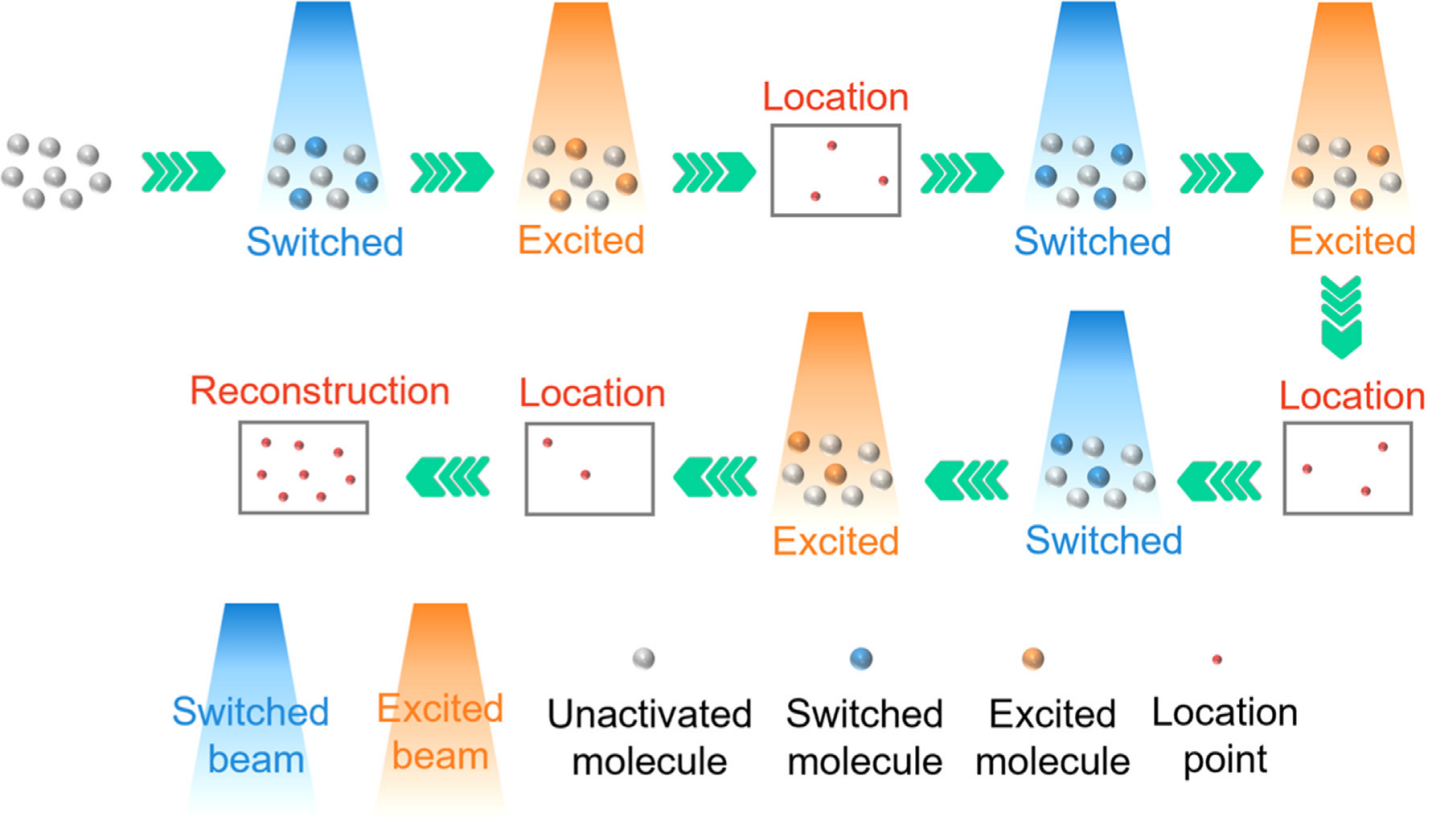
Schematic of STORM.

STORM has a similar operating principle to PALM. However, compared with PALM, STORM improves the rate of super-resolution image acquisition. The photobleaching of active fluorophores in PALM is considered an additional time-consuming step. Thus, STORM replaces this step with light-switchable fluorophores, which can be activated (turned on) and deactivated (turned off) at will (Fig. 9). This improvement eliminates the requirement of continuous photobleaching and fluorescent group activation, thus reducing the total collection time. Both PALM and STORM can be easily implemented in any laboratory equipped with TIRF-M or wide-field fluorescence microscope, which not only simplifies the experimental process but also increases accuracy and sensitivity. Importantly, benefiting from the high spatial resolution, super-resolution microscopy techniques enable for an in-depth investigations of the characteristics and early morphology of LLPS droplets.

**FIG. 10.**
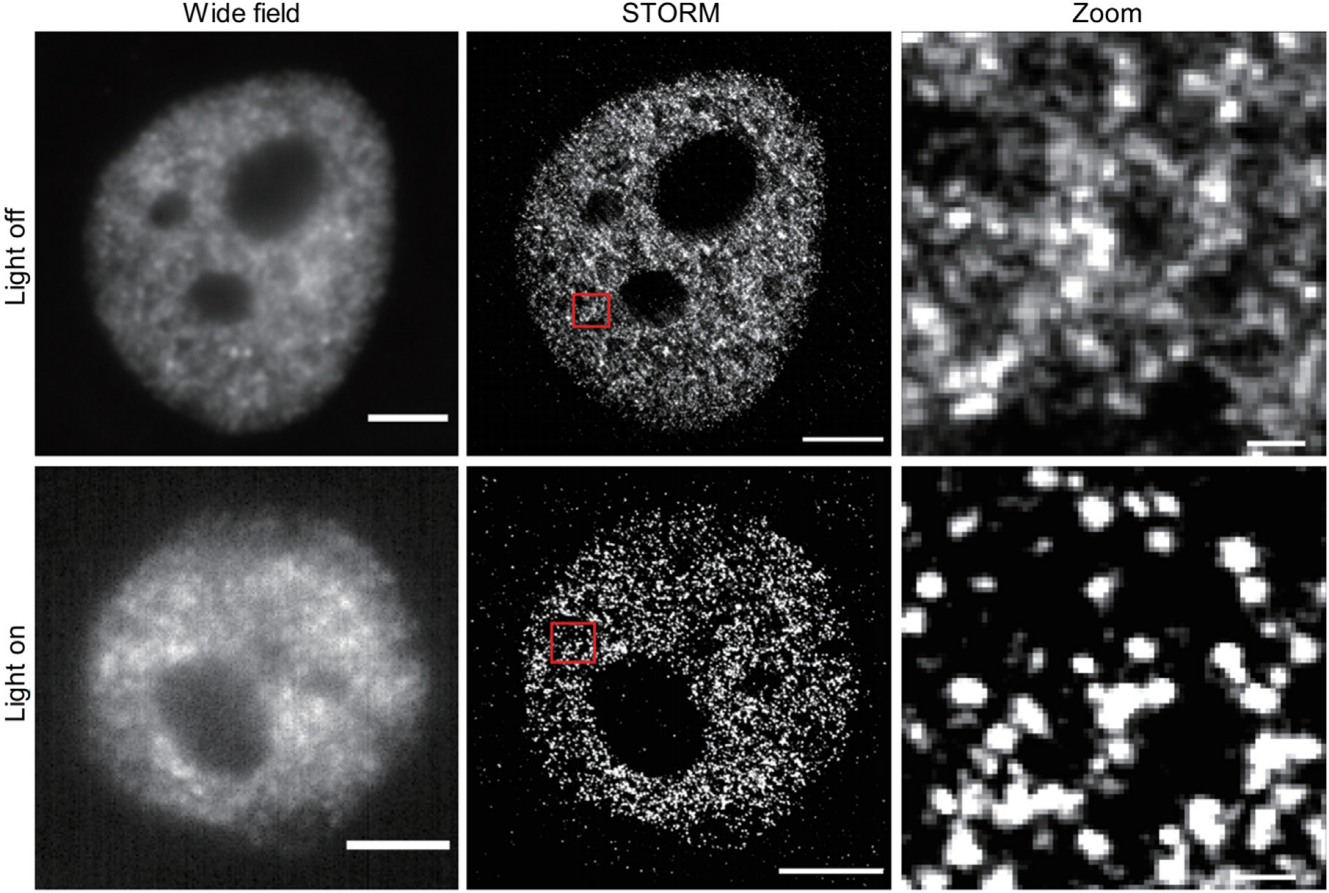
Study LLPS with the super-resolution microscope. Representative wide field and STORM images of CRY2-RPB1 in blue light on and off states. Scale bar = 2 *μ*m. Zoomed scale bar = 100 nm. [Reproduced with permission from Zhang *et al.*, Biochem. Biophys. Rep. **8**(1), 2–13 (2022). Copyright 2022 Authors, licensed under a Creative Commons Attribution 4.0 International License.^99^]

Recently, Zhang *et al.* presented a comprehensive protocol regarding the use of super-resolution-microscopy to study LLPS (Fig. 10). Using RNA Pol II as an example, they described the morphology and quantified the number of molecules in LLPS droplets in cells. This protocol offers significant assistance in employing super-resolution microscopy to study the wide variety of phase separations currently known and can drive the understanding of LLPS mechanisms to higher and deeper levels. Ulianov *et al.*, using STORM, investigated the effects of 1,6-hexanediol-mediated disruption/modulation of LLPS on higher-order chromatin organization in living cells.^97^ Their study showed that 1,6-hexanediol treatment resulted in an enlargement of nucleosome clutches and a more even distribution in the nuclear space. The LLPS phenomenon of *par*s and ParB in bacteria was studied by Eigen and Rigler.^98^ They found that *par*s and ParB associate to form nanoscale droplets. Importantly, ParB molecules can diffuse between different droplets. These results describe a novel mechanism of activity, which has been difficult to achieve with conventional microscopy.

The rapid advancement of super-resolution microscopy has enabled the visualization of subcellular components and processes, making it particularly suitable for the study of LLPS. However, super-resolution microscopy techniques achieve enhanced spatial resolution at the expense of temporal resolution. Although super-resolution microscopy is highly efficient at capturing the morphology of nanoscale LLPS droplets, it is difficult to obtain high-speed dynamics of droplets.

Structured illumination microscopy (SIM) is a super-resolution microscopy technique that utilizes interference patterns created between the sample and the illumination pattern.^100–103^ In SIM, patterned light is projected onto the sample, interacting with its structures to produce a moiré pattern. This pattern contains high-frequency information beyond the diffraction limit of the microscope, enabling high-resolution image acquisition. To generate the final image, the illumination pattern is shifted and repeated multiple times with slight variations, and the resulting images are computationally combined. SIM offers several advantages over other super-resolution techniques such as PALM/STORM. It can enhance resolution, increase image contrast, and reduce phototoxicity and photobleaching. Moreover, SIM is compatible with live-cell imaging and can be used with standard fluorescent dyes. The numerous benefits of the SIM technique have made it a valuable tool for studying LLPS. As a result, the SIM technique has been widely applied in research on LLPS, enabling researchers to gain insights into the mechanisms of their early formation. Levone *et al.* conducted a quantitative study of γH2AX nanofoci in WT and FUS-KO cells using 3D-SIM technology. The study findings suggest that defective DNA damage repair in FUS-KO cells is linked to inefficient clustering of γH2AX nanofoci.^104^ Recently, Wang *et al.* introduced an accelerated reconstruction algorithm for SIM, which exhibits an 80-fold improvement in reconstruction speed.^105^ This algorithm effectively accelerates image processing without compromising spatial resolution or slicing ability. With its high spatial and temporal resolution, this technique holds great promise as an important tool for studying the mechanisms of early LLPS formation in the future.

### E. Fluorescence correlation spectroscopy (FCS)

Another method for measuring the kinetics of fluorescently labeled molecules is fluorescence correlation spectroscopy (FCS), which is a single-molecule fluorescence detection technique.^98^ FCS calculates the time correlation of fluorescence intensity fluctuations and the decay time, which indicates the molecule diffusing speed, can be extracted. As an optical technique that allows quantitative analysis, FCS can provide: (1) the average number of molecular species within the FCS volume element and their respective molar concentrations; (2) protein equilibrium dissociation rate and the homo- or hetero-molecular stoichiometric ratios in LLPS droplets; (3) the droplet diffusion coefficients and corresponding hydrodynamic radius. Thus, FCS has been widely used in studies of single molecule diffusion, conformational changes, molecular binding/dissociation equilibria, and biochemical reaction kinetics.^43^ Moreover, FCS has proven to be a valuable tool for studying phase separation processes in lipid bilayers^106,107^ and has been applied to study biomolecular LLPS both *in vivo* and *in vitro*.^108^

**FIG. 11.**
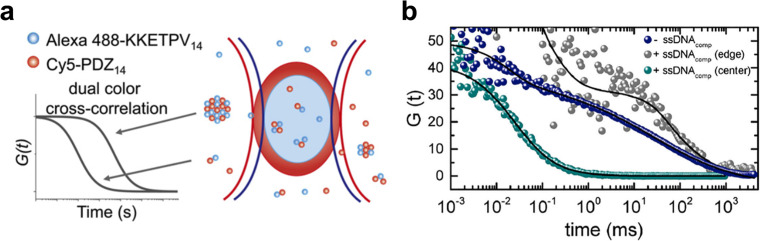
(a) Detection scheme for dcFCCS. Alexa 488-KKETPV_14_ (blue dots) and Cy5 PDZ_14_ (red dots) molecules freely diffuse through the confocal probing volume of 488 nm (blue oval) and 640 nm (red oval) lasers. Only the heterocomplex containing both Alexa 488 and Cy5 fluorophores contribute to the dcFCCS curve, and its relaxation time correlates with the size of the complex. [Reproduced with permission from Peng *et al.*, Proc. Natl. Acad. Sci. U.S.A. **117**(44), 27124–27131 (2020). Copyright 2020 Authors, licensed under a Creative Commons Attribution 4.0 International License.^109^] (b) FCS autocorrelation curves are measured for labeled PLL before and after the addition of ssDNA_comp_. [Reproduced with permission with permission from Shakya *et al.*, Biophys. J. **115**(10), 1840 (2018). Copyright 2018 Elsevier.^110^]

Recently, Peng *et al.* captured the formation of nanoscale condensates using a dual color fluorescence cross correlation spectroscopy (dcFCCS) method [Fig. 11(a)]. They quantified the condensates' properties such as size and growth rate. They noted that these nano-sized condensates could hardly be accurately observed using conventional fluorescence microscopy. However, they may play important roles in various cellular processes. Thus, it is imperative to employ dcFCCS, which is a simple and robust quantitative tool to study them in detail. Shakya *et al.* studied junction LLPS after complexation of DNA with cationic polypeptides utilizing FCS [Fig. 11(b)]. They found that it was not merely the charge density but the local flexibility of the DNA that determined the LLPS. In addition, free nucleotide triphosphates can effectively promote the LLPS of polypeptide dsDNA complexes.

### F. Fluorescence recovery after photobleaching (FRAP)

Confocal microscopy enables visualization of the LLPS droplet formation process. However, due to sensitivity and diffraction limitations, it remains challenging to obtain molecular dynamics within the droplet from confocal images. Fluorescence recovery after photobleaching (FRAP), an imaging technique based on confocal microscopy, appears as a valuable tool to characterize molecular dynamics in LLPS droplets.^111^ FRAP is a widely used method for determining the occurrence of LLPS and can also be used to quantify the dynamics within LLPS condensates.^112^

**FIG. 12.**
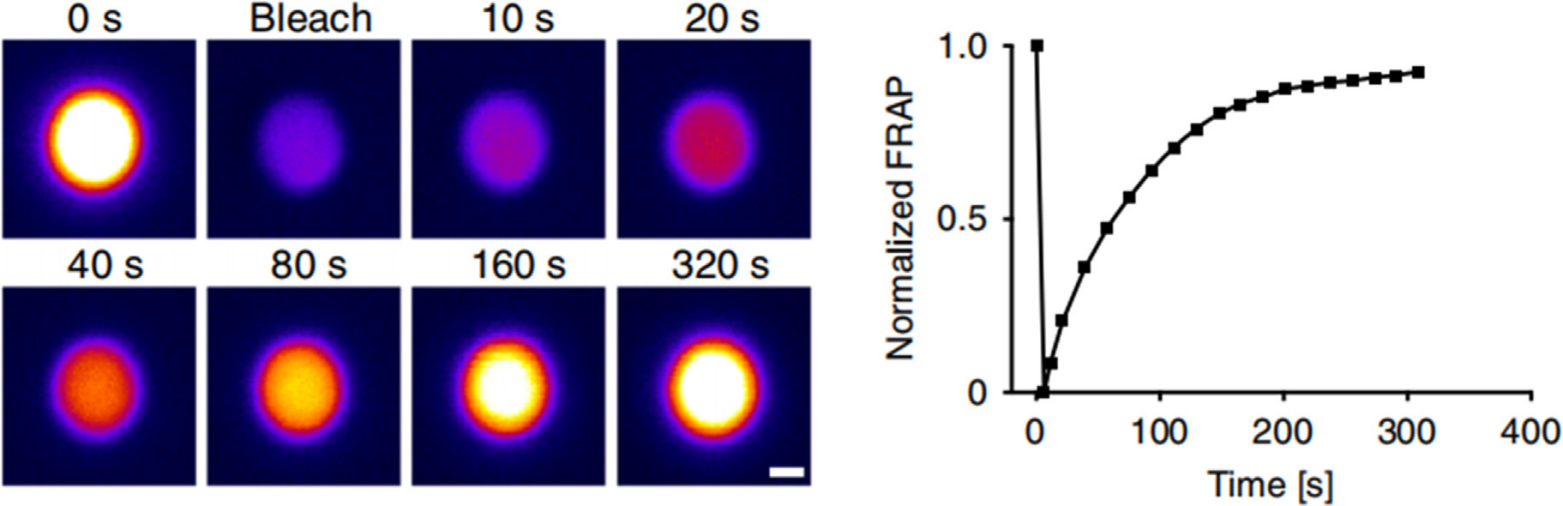
Study the dynamic nature of tau droplets with FRAP. Fluorescence bleaching recovery images of a single tau protein droplet. (LEFT) fluorescence recovery rate curve (right). [Reproduced with permission from Kanaan *et al.*, Nat. Commun. **11**, 2809 (2020). Copyright 2020 Authors, licensed under a Creative Commons Attribution 4.0 International License.^79^]

In the FRAP experiment, a stationary laser spot is typically used to photobleach the fluorescence of the sample, followed by imaging of the area after a certain time. Because of the mobility of molecules in the sample, fluorescence in the prebleached area partially recovers. The recovery speed can be used to calculate the molecular exchange rate near the bleaching area.^113^ For example, Zhu and Gu *et al.* found that the fluorescence intensity of RBGD2- and RBGD4-containing granules recovered rapidly within 20–50 s after photobleaching, suggesting that these structures are highly dynamic.^32^ This experiment confirmed that RBGD2 and RBGD4 act as important LLPS proteins in response to heat stress in plant cells. Kang *et al.* used FRAP to investigate whether FXR1 exhibits a liquid-like condensate state and characterized the dynamic recombination and exchange kinetics within its liquid-like condensate.^69^ Furthermore, the dynamic properties of mitochondria-associated droplets in mammalian oocytes were characterized by FRAP.^70^ FRAP experiments on LLPS droplets of tau protein *in vivo* and *in vitro* confirmed the fluidity and dynamic reorganization ability of the droplets (Fig. 12). In Arabidopsis, light-induced LLPS droplets were characterized for their dynamic recombination properties using FRAP.^29^ When LLPS droplets undergo a liquid-like to solid-like phase transition, the mobility of the molecules within the droplet is reduced. This transition can be monitored using FRAP, with a decrease in fluorescence recovery rate indicating reduced molecular mobility. Solid-like structures formed by proteins undergoing phase transitions have been implicated in the development of neurodegenerative diseases, such as Alzheimer. These diseases are characterized by the formation of protein aggregates, which often appear solid-like. Using FRAP to monitor the dynamics of protein molecules within these aggregates can provide insight into their role in disease pathology. In summary, FRAP can be used to not only validate the droplet formation but also to perform quantitative analysis of LLPS-related protein properties.

### G. Capillary flow experiments

It is important to note that while these microscopes mentioned above are useful for characterizing LLPS, they may not be suitable for high-throughput experiments. Capillary flow experiment (Capflex) could potentially measure not only droplet size, kinetics, and thermodynamics of droplet formation and maturation but also describe the affinity of proteins/peptides undergoing LLPS for various interactions.^114^ When the sample is injected into the capillary tube in FIDA 1 and maintained in a temperature-controlled chamber, the sample passes through the LLPS and reaches the detector after a time delay for flow and pressure control. As each droplet passes, a signal peak is recorded. The baseline intensity level of the signal in this method allows the protein concentration in the light/dilute phase. This method is suitable for measuring not only yellow fluorescent protein (YFP) or Alexa488 dyes but also intrinsic proteins. In addition, only nanomolar (nM) protein concentrations are required for the measurement. For example, the text discusses the characterization of human dead box decapping enzyme-4 and LLPS ssDNA/RP3 of the cohesive system using Capflex to study LLPS of α-synuclein and abnormal liquid–solid phase transition.^114^ In addition to quantitative measurements, the formation of thioflavin-LLPS-positive amyloid aggregates was accompanied by a decrease in dilution phase concentration.

## IV. PROBES IN LLSP STUDY

To image proteins and droplets of LLPS under fluorescent microscopy, it is essential to label the target protein or molecules with fluorescent probes. The most commonly used fluorescent probes include fluorescent proteins and organic dyes.

To observe dynamic LLPS processes in living cells, LLPS proteins are usually fused with fluorescent proteins or SNAP/Halo tags, which can further be labeled with small molecule fluorescent probes via click chemistry. Some reported fluorescence proteins used in LLPS studies include mScarlet,^115^ mEGFP (mutation in EGFP),^28^ mClover3,^70^ and DsRed.^29^ Chandra *et al.* studied the LLPS of NUP98–HOXA9 (NHA9), one of the NUP98 fusion oncoproteins (FO) by expressing mEGFP-NHA9 and truncated protein mEGFP-NHA9-ΔDNA in HEK293T cells.^28^ Their studies revealed that the number of mEGFP-NHA9 condensates is positively associated with its nuclear concentration, indicating that the LLPS NHA9 is dependent on multivalent phenylalanine-glycine (FG) motifs and interactions of the HOXA9 homeodomain with DNA. Moreover, CRY2-DsRed photobodies demonstrate dynamic reversibility in response to light/dark cycles in the nucleus of *Arabidopsis* protoplast and CRY2 undergoes LLPS *in vivo* upon exposure to blue light.^29^

While expressing fluorescence proteins in cells is convenient and highly efficient, their over expression and multimerization may impact LLPS. Therefore, using Halo-Tag, a modified haloalkane dehalogenase that covalently binds to synthetic ligands with high specificity and efficiency,^116^ can be a better choice. Halo-Tag is widely used for site-specific labeling of proteins, fluorescent labeling of living cell proteins, affinity treatment, and solid phase adsorption. Recently Zhang *et al.* employed single-molecule tracking to measure the dynamics of heat-shock transcription factor 1 (HSF1) by Halo-Tag into the HSF1 gene locus and labeling it with Halo-JF549 dye.^117^ They observed that HSF1 molecule motion was confined upon heat shock owing to LLPS while in the absence of heat shock, HSF1 molecule motion remained highly dynamic. Colocalization between HSF1 and heat-shock-protein (HSP) genes indicates that HSF1 condensate interacts with HSF1-target genes through multivalent and weak interactions in response to heat shock.

There are also some small-molecule fluorescent probes available for LLPS live-cell imaging. Shao *et al.* developed a kind of SG small-molecule probe (E)-3-methyl-6-(piperazin-1-yl)-2–(2–(2,3,6,7-tetrahydro-1H,5H-pyrido[3,2,1-ij]quinolin-9-yl)vinyl)benzo[d]thiazol-3-ium trifluoroacetate (TASG), which not only binds to the core SG protein G3BP1 and its complexes but also exhibits minimal fluorescence in the absence of SG.^118^

Immunostaining is also used to image proteins and droplets of LLPS in fixed cells and tissue sections using antibodies. In addition, Congo Red,^119^ Thioflavin S/T,^120,121^ (trans, trans)-1-bromo-2,5-bis-(4-hydroxy)styrylbenzene (K114),^122^ and Amylo-Glo^123^ are used to detect amyloid inclusions *in situ* and monitor the maturation of assembled amyloid fibrils. To summarize, while immunostaining involves complex processes and lacks real-time monitoring capabilities, it offers advantages such as high affinity, high sensitivity, and high accuracy.

To remodel LLPS *in vitro*, recombinant proteins are expressed using procaryotic expression system and labeled with amine-reactive dyes. For instance, Zhang *et al.* labeled Δtau187 with Alexa Fluor 488 NHS Ester and studied the droplet formation process in the presence of poly(A) RNA.^64^ In addition, both Oregon Green 488-RBGD2 and Alexa Fluor 350-RBGD4 can form droplets in a concentration-dependent manner, while RBGD2 and RBGD4 also co-phase separate and colocalize when mixed together.^32^ In brief, this method can be employed not only to explore the conditions that induce LLPS, but also to track the dynamic process of LLPS *in vitro*.

In the context of LLPS, using fluorescent probes presents two main challenges. The first challenge concerns the effect of viscosity on the fluorescence quantum yield of the probe.^124^ Fluorescence quantum yield is a measure of the efficiency of a probe in converting absorbed light into emitted fluorescence. However, the viscosity of the solution in which the probe is present can affect its fluorescence quantum yield. In LLPS, the viscosity of the liquid-like droplets can differ from that of the surrounding environment, leading to changes in fluorescence quantum yield that can impact the accuracy of measurements obtained using fluorescent probes. The second challenge is associated with covalent labeling of probes. Covalent labeling involves attaching a fluorescent molecule to a protein or other molecule of interest through a chemical bond. However, covalent labeling can alter the properties of the labeled molecule, potentially affecting its behavior within the LLPS droplets. This can lead to artifacts in experimental measurements and affect the accuracy of conclusions drawn from the data. Overall, the use of fluorescent probes in the context of LLPS requires careful consideration of the potential challenges associated with their use, such as the effect of viscosity on fluorescence quantum yield and potential artifacts associated with covalent labeling. By addressing these challenges, researchers can obtain accurate measurements and draw reliable conclusions from their experiments.

## V. SUMMARY AND OUTLOOK

The LLPS-induced cellular condensates possess multiple biochemical and biological functions along with unique biophysical properties.^125^ In order to gain a comprehensive understanding of the molecular composition and biological functions of these condensates, a range of techniques are employed, including fluorescent protein engineering, immunostaining, optical imaging, and others.^60,125,126^

When conducting LLPS studies, it is important to carefully select the appropriate microscopy based on the research object, and correlative optical imaging can be used if necessary (Table I). For example, while the DIC microscope is highly efficient in characterizing the morphology of LLPS droplets, it lacks specificity and cannot resolve intracellular LLPS. Combining confocal microscopy technology with labeling techniques can overcome this limitation, albeit at the cost of temporal resolution. TIRF-M provides single molecule sensitivity but is limited to imaging near the surface, and the size of droplets is often smaller than the optical diffraction limit in the early stage. While super-resolution microscopy techniques such as PALM and STORM can break this limitation, it is challenging to characterize dynamics using these methods. On the other hand, FRAP and FCS are useful tools for studying the diffusion dynamics of the droplets.

**TABLE I.** Comparison of optical imaging methods in LLPS studies.

Method	Advantages	Disadvantages	References
DIC	High contract; Do not need fluorescent tags; High acquisition rate	Lack specificity; Challenging for intracellularly imaging; Poor spatial resolution.	20,77–79
Wide-field	Easy to implement; Low phototoxicity; High acquisition rate	Poor signal-to-noise ratio; Lack axial resolution; Poor spatial resolution	72, 76
Confocal	High signal-to-noise ratio; High axial resolution	High phototoxicity; Slow acquisition rate	20, 29, 32, 69, 70, 77, 79
Spinning disk confocal	High acquisition rate; Minimal background; Reduced phototoxicity	Expensive and complex to implement	82
TIRF	High signal-to-noise ratio; Single-molecule sensitivity; Low phototoxicity; High acquisition rate	Limited imaging depth; Lack axial resolution	87, 88
SMLM	High signal-to-noise ratio; Single-molecule sensitivity; High spatial resolution	Slow acquisition rate; High phototoxicity; Images require postprocessing	97, 99
SIM	Single-molecule detection; High spatial resolution	Slow acquisition rate; High phototoxicity;	104, 105
FCS	Single-molecule sensitivity; Quantitative analysis	Lack morphological information.	108–110
Capflex	High sensitivity High throughput High precision	Limited to certain types of analytes; Limited resolution	114

Choosing the appropriate optical imaging method for LLPS studies involves several factors, including sample size and thickness, imaging speed, and potential phototoxicity and photobleaching effects. For example, DIC, wide-field microscopy, and confocal microscopy are suitable for imaging thicker samples, while TIRF microscopy is ideal for thin samples. High-resolution imaging can be achieved using techniques such as SMLM, SIM, and TIRF. Some imaging techniques may cause phototoxicity or photobleaching, which can affect the sample over time. Methods, such as FCS and Capflex imaging, are less likely to cause such issues. Cost is also a consideration when selecting an imaging technique. In summary, the choice of the imaging method for LLPS studies will depend on various factors. It may be necessary to use multiple techniques to obtain a comprehensive understanding of LLPS behavior.

In recent years, real-time 3D single molecule tracking has emerged as a powerful tool for studying molecular dynamical interactions, thanks to its high spatiotemporal precision, high sensitivity, and large imaging field.^127–140^ It can be expected that real-time 3D single molecule tracking will play an increasingly important role in future LLPS studies. Light-sheet microscopy, on the other hand, offers a field of view, high imaging speed, and low light dose, making it a promising option for long-term observation.^141–146^ Furthermore, the rapid development of image data processing using machine learning also inspires that it can be more involved in the LLPS studies.^147–153^ For example, it may be possible to extract the composition information from the image data with the aid of machine learning.

## Data Availability

Data sharing is not applicable to this article as no new data were created or analyzed in this study.
